# DACH1 inhibits SNAI1-mediated epithelial–mesenchymal transition and represses breast carcinoma metastasis

**DOI:** 10.1038/oncsis.2015.3

**Published:** 2015-03-16

**Authors:** F Zhao, M Wang, S Li, X Bai, H Bi, Y Liu, X Ao, Z Jia, H Wu

**Affiliations:** 1 School of Life Science and Biotechnology, Dalian University of Technology, Dalian, China; 2 School of Life Science and Medicine, Dalian University of Technology, Panjin, China

## Abstract

Epithelial–mesenchymal transition (EMT) has a major role in cancer progression and metastasis. However, the specific mechanism of transcriptional repression involved in this process remains largely unknown. Dachshund homologue 1 (DACH1) expression is lost in invasive breast cancer with poor prognosis, and the role of DACH1 in regulating breast cancer metastasis is poorly understood. In this study, significant correlation between the expression of DACH1 and the morphology of breast cancer cells was observed. Subsequent investigation into the relationship between DACH1 and EMT showed that overexpression of DACH1 in ZR-75-30 cells induced a shift towards epithelial morphology and cell–cell adhesion, as well as increased the expression of the epithelial marker E-cadherin and suppressed cell migration and invasion. In contrast, silencing DACH1 in MCF-7 and T47D cells disrupted the epithelial morphology and cell–cell contact, reduced the expression of E-cadherin, and induced cell migration and invasion. DACH1 also specifically interacted with SNAI1, but not SNAI2, to form a complex, which could bind to the E-box on the E-cadherin promoter in an SNAI1-dependent manner. DACH1 inhibited the transcriptional activity of SNAI1, leading to the activation of E-cadherin in breast cancer cells. Furthermore, the level of DACH1 also correlated with the extent of metastasis in a mouse model. DACH1 overexpression significantly decreased the metastasis and growth of 4T1/Luc cells in BALB/c mice. Analysis of tissue samples taken from human breast cancers showed a significant correlation between the expression of DACH1 and E-cadherin in SNAI1-positive breast cancer. Collectively, our data identified a new mechanistic pathway for the regulation of EMT and metastasis of breast cancer cells, one that is based on the regulation of E-cadherin expression by direct DACH1–SNAI1 interaction.

## Introduction

Breast cancer is the most common malignancy and the first leading cause of cancer-related death in females worldwide.^1^ Most breast cancer-related deaths are caused by highly metastatic tumours, in which the primary tumour cells would move through the blood capillaries or draining lymphatic vessels to new organ sites.^2, 3^ To provide further insight that will enable the development of new therapeutic strategies, it is crucial to elucidate the molecular mechanisms that promote the invasive and metastatic properties of breast cancer cells. Recent studies have shown that aberrant activation of epithelial–mesenchymal transition (EMT) has been implicated in this process.^4, 5^

EMT endows cells with migratory and invasive properties, eventually leading to stem cell properties and immunosuppression.^6, 7^ During this process, epithelial tumour cells may lose their characteristics, including cell–cell adhesion and polarity accompanied by cytoskeleton rearrangements. They may acquire a migratory behaviour, allowing them to move away from their microenvironment and into surrounding or remote locations.^8, 9^ Sometimes, cells that undergo EMT can transiently re-acquire an epithelioid phenotype by reverse mesenchymal–epithelial transition.^10, 11, 12^ In most epithelial cancers, loss of E-cadherin gene or protein expression is frequently found in tumour cells that undergo EMT. Hence, E-cadherin is emerging as one of the hallmarks of EMT.^6, 12, 13^ E-cadherin-mediated cell–cell adhesion complexes are anchored to the actin cytoskeleton via its cytoplasmic domain and *β*-catenin and *α*-catenin.^13, 14, 15^ EMT is triggered by multiple extracellular stimuli. Among these extracellular cues, transforming growth factor-*β* has a predominant role.^6, 12^ In addition, some zinc-finger transcription factors, including SNAI1,^16^ SNAI2,^17^ ZEB1^18^ and ZEB2,^19^ have also been found to promote EMT through direct binding to the E-box of the E-cadherin promoter.

In the past few years, SNAI1 has emerged as one of the important classical EMT transcription factors in cancer research.^20^ SNAI1 repression of E-cadherin involves the direct recruitment of a repressor complex formed by the corepressors SIN3A and HDAC1/2.^21^ Some histone modifiers such as the methyltransferases G9a^22^ and Suv39H1^23^ are associated with SNAI1 activity in human breast cancer. The repressive transcriptional activity of SNAI1 can also be modulated by other factors, such as ALX1^7^ and AIB1.^5^ Furthermore, SNAI1 can also bind to its own promoter and repress its own expression.^24^ In addition to the classical EMT factors, recent studies have uncovered many novel EMT-related transcription factors, such as FOXQ1^25^ and GLI1.^26^ These studies indicate that EMT is modulated by many classical and non-classical factors. Therefore, finding and understanding the regulation of novel factors would provide important insight into the molecular mechanisms implicated in EMT.

The *Dachshund* gene, initially identified as essential for Drosophila eye, limb, brain and gonadal development, encodes a key component of the retinal determination gene network in Drosophila eye development.^27, 28^ Recent studies have demonstrated an important role for the human Dachshund homologue 1 (DACH1) in tumourigenesis, particularly those of the breast, prostate, ovarian, brain and lung.^28, 29, 30^ DACH1 is expressed in normal epithelium but its expression is significantly reduced in breast, prostate and endometrial cancer.^31, 32, 33^ DACH1 regulates its target genes, in part by interacting with transcription factors, including HOXA9, p53, SMADs, SIX and others,^28, 34, 35, 36, 37^ and also by binding to the DNA via AP-1, NF-κB and Forkhead binding sites.^38, 39^ The role of DACH1 in the inhibition of oncogene-induced cellular migration and metastasis is well established in breast cancer cells.^38, 40^ However, the molecular mechanism by which DACH1 regulates metastasis and EMT remains largely unknown.

In this study, we found that DACH1 overexpression suppressed EMT in ZR-75-30 cells, whereas DACH1 knockdown induced EMT in MCF-7 and T47D cells. Subsequent investigation showed that DACH1 suppressed EMT and metastasis by increasing the expression of E-cadherin. We also proposed that DACH1 interacted with SNAI1 at the E-box of E-cadherin promoter and inhibited the transcriptional activity of SNAI1, leading to the activation of E-cadherin in breast cancer cells.

## Results

### DACH1 expression is associated with the morphology of breast cancer cells

**Figure 1:**
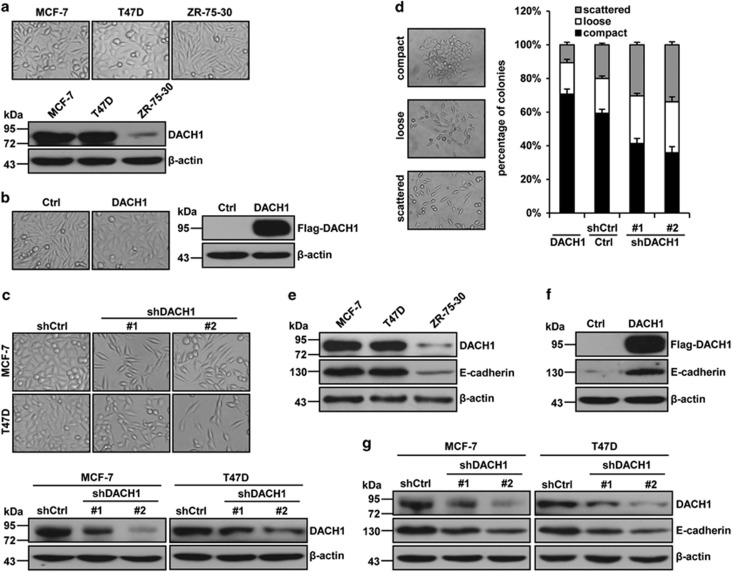
DACH1 regulates breast cancer cellular morphology. (**a**) Phase-contrast microscopic images of the human breast cancer cell lines MCF-7, T47D and ZR-75-30 (top), and western blot analysis of DACH1 expression in these cells (bottom). (**b**) Phase-contrast microscopic images showing the effect of DACH1 overexpression on the cell–cell adhesion and morphology of ZR-75-30 cells (top). Western blot analysis of DACH1 overexpression in ZR-75-30 cells (bottom). Cells were transfected with pcDNA3.1-3 × lag-DACH1 or pcDNA3.1-3 × Flag vector and selected for 5 days in the presence of G418. (**c**) Phase-contrast microscopic images showing the changes in cell–cell contact and morphology of MCF-7 and T47D cells as a result of DACH1 knockdown (top). Western blot analysis of DACH1 knockdown in MCF-7 and T47D cells (bottom). Cells were transfected with shDACH1#1, shDACH1#2 and shCtrl (pRNAT-U6.1 vector) and selected for 5 days in the presence of Hygromycin B. (**d**) Cell colony scattering assay showing the effect of DACH1 overexpression or knockdown on the scattering of MCF-7 cells. Cells were transfected with pcDNA3.1-3 × Flag-DACH1 or shDACH1 (pRNAT-U6.1 vector) and selected for 5 days in the presence of G418 or Hygromycin B, and then plated in a 10-cm dish at a very low density to allow them to form small colonies. Morphology and number of colonies were analyzed 6 days after plating. According to the extent of cell–cell adhesion, colonies were categorized into three forms: compact, loose and scattered. (**e**) Expression of DACH1 and E-cadherin in breast cancer cells. (**f**) Effect of DACH1 overexpression on E-cadherin in ZR-75-30 cells. (**g**) Effect of DACH1 knockdown on E-cadherin expression in MCF-7 and T47D cells.

Among the three different human breast cancer cell lines that were investigated, high level of DACH1 expression was displayed by MCF-7 and T47D cells, which also displayed epithelioid morphology and prominent cell–cell contact (Figure 1a). ZR-75-30 cells, which showed low level of DACH1 expression, displayed mesenchymal-like morphology and lesser cell–cell adhesion. This suggested that DACH1 may have an important role in the regulation of cell morphology. To shed more light on this speculation, ZR-75-30 cells were transfected with DACH1, whereas MCF-7 and T47D were transfected with shRNA (shDACH1#1 and shDACH1#2) and the changes in cell morphology were examined. Surprisingly, overexpression of DACH1 in ZR-75-30 cells dramatically increased cell–cell adhesion and changed the morphology of the cells from arciform and spindle shape to oval shape, more like epithelial cells (Figure 1b). In contrast, depletion of DACH1 in MCF-7 and T47D cells greatly reduced cell–cell contact and resulted in more spindle-shaped cells (Figure 1c). In order to quantify the effect of DACH1 on cell–cell adhesion, we carried out a colony scattering experiment to evaluate the ability of the cells to form scattered colonies. Cells that overexpressed DACH1 formed about 1.2-fold more compact colonies and 2-fold fewer scattered colonies compared with control cells, whereas cells with knockdown of DACH1 formed ~1.5-fold fewer compact colonies and 1.5-fold more scattered colonies compared with control cells (Figure 1d).

Next, we sought to determine whether the epithelial marker E-cadherin has a part in this DACH1-induced morphological change and colony formation observed for these breast cancer cells. Surprisingly, DACH1 and E-cadherin were uniformly expressed at background levels in all breast cancer cell lines (MCF-7, T47D and ZR-75-30 cells) tested (Figure 1e). To further investigate the possible role of DACH1 in the regulation of E-cadherin expression, the effect of DACH1 overexpression on E-cadherin protein expression in ZR-75-30 cells was examined, and the result showed that overexpression of DACH1 increased the expression of E-cadherin (Figure 1f). In contrast, depletion of DACH1 with shDACH1 in MCF-7 and T47D cells suppressed the expression of E-cadherin (Figure 1g). Taken together, these data indicated that DACH1 may be crucial for breast cancer cells to maintain an epithelial morphology and cell–cell contact.

### DACH1 regulates cell invasiveness and EMT markers

**Figure 2:**
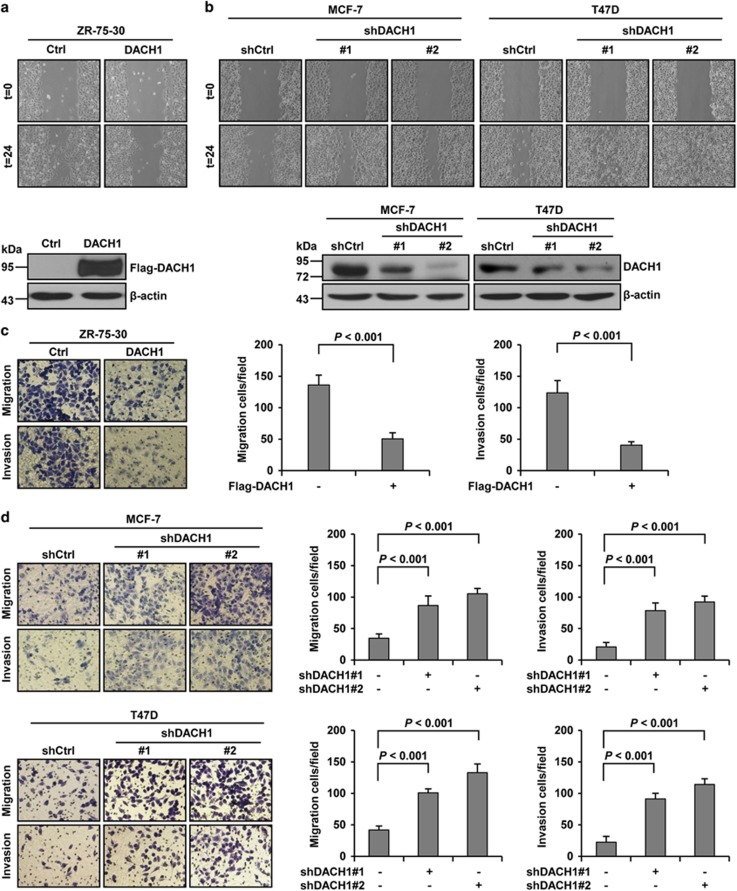

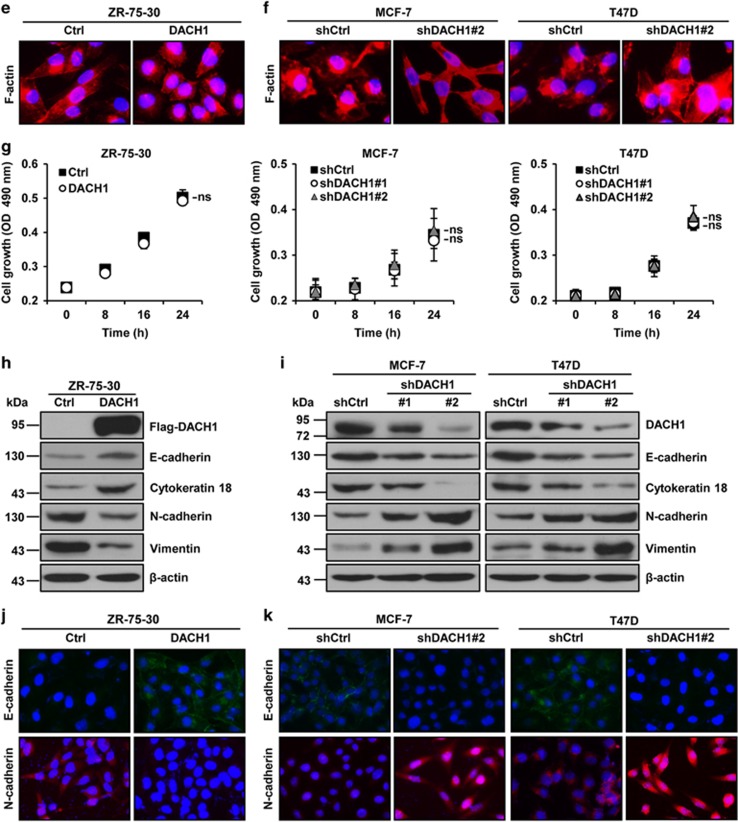
DACH1 modulates breast cancer cell motility, invasion and EMT markers. (**a** and **b**) Scratch wound-healing assay assessing the effects of DACH1 overexpression and knockdown on the motility of ZR-75-30, MCF-7 and T47D cells. ZR-75-30 cells were transfected with pcDNA3.1-3 × Flag-DACH1 or pcDNA3.1-3 × Flag vector and selected for 5 days in the presence of G418; MCF-7 and T47D cells were transfected with shDACH1#1, shDACH1#2 and shCtrl (pRNAT-U6.1 vector) and selected for 5 days in the presence of Hygromycin B. The cells were then plated in six-well plates. After 12  h of incubation, confluent cells were carefully scratched with a sterile 200-μl pipette tip and incubated for another 24  h before evaluation. (**c** and **d**) Transwell migration and invasion assays evaluating the effects of DACH1 overexpression and knockdown on the cellular motility and invasion ability of ZR-75-30, MCF-7 and T47D cells. Cell migration and invasion assays were performed in 24-well chambers without or with Matrigel. Cells (1 × 10^6^ per well) were transferred to the upper chamber. After 24  h of incubation, the migrating and invading cells on the lower surface of the filter were stained and counted (left). Cells were transfected as in (**a** and **b**) and then plated in the upper chamber. The bar graphs show the number of migrating and invading cells for each category of cells (right). (**e** and **f**) F-actin immunofluorescence showing the effects of DACH1 overexpression and knockdown on the motility of ZR-75-30, MCF-7 and T47D cells. (**g**) MTT assay assessing the effects of DACH1 overexpression and knockdown on cell proliferation in ZR-75-30, MCF-7 and T47D cells. Cells were transfected as in (**a** and **b**) before being plated in 96-well plates and then subjected to MTT assay within 24 h. (**h** and **i**) Western blots showing the effects of DACH1 overexpression and knockdown on the expression levels of E-cadherin, Cytokeratin 18, N-cadherin and Vimentin in ZR-75-30, MCF-7 and T47D cells. (**j** and **k**) Immunofluorescence showing the effects of DACH1 overexpression and knockdown on the expression levels of EMT markers in ZR-75-30, MCF-7 and T47D cells. Cells were transfected as in (**a** and **b**) and then cultured in six-well chamber coverslips. After 24  h, the cells were fixed and immunostained with anti-E-cadherin and anti-N-cadherin antibodies and then incubated with secondary antibodies (Alexa Fluor 568, Life Technologies, Carlsbad, CA, USA). Nuclear proteins were stained with DAPI (4′,6-diamidino-2′-phenylindole dihydrochloride). Data are presented as means±s.d.s. Significance was determined using the Bonferroni test (*P*<0.05, significant ns, not significant).

To extend our analysis of the role of DACH1 in cell motility, we used scratch wound-healing and transwell assays to demonstrate the migration and invasion capacity, respectively, of breast cancer cells. Overexpression of DACH1 in ZR-75-30 cells significantly inhibited cell motility and reduced the number of cells that migrated through the Matrigel-coated membrane, compared with control cells (Figures 2a and c). In contrast to ZR-75-30 cells, we generated MCF-7 and T47D cells that expressed shRNA (shCtrl or shDACH1) to further test the effect of DACH1 on cell migration and invasion. The result showed that knockdown of DACH1 markedly enhanced the migration, and increased the capacity of cells to traverse the Matrigel-coated membrane, compared with control cells (Figures 2b and d). In addition, the organization of actin cytoskeleton in the cells with DACH1 overexpression or knockdown was also compatible with its migratory behaviour. In ZR-75-30 cells that overexpressed DACH1, F-actin was not organized in abundant stressed fibres, compared with control cells (Figure 2e). In MCF-7 cells, F-actin was also not organized in abundant stressed fibres, but silencing of DACH1 resulted in abundant but organized stress actin fibres (Figure 2f). These results suggested that DACH1 may have a key role in the suppression of mobility and invasiveness, which may possibly be connected to its effect on the organization of the actin cytoskeleton of breast cancer cells. The reduced motility and invasiveness of DACH1 was not due to impaired viability of the cells because cell proliferation (MTT assay) was neither suppressed nor increased by overexpression and knockdown of DACH1 within the duration of the experiment (24 h) (Figure 2g). Based on the observation that DACH1 could maintain an epithelial morphology, increase the expression of epithelial markers E-cadherin, and suppress cell migration and invasion, we speculated that DACH1 might block the transition from epithelial to mesenchymal phenotype in these breast cancer cells. To test this hypothesis, the expression levels of epithelial and mesenchymal markers were examined. DACH1 overexpression greatly increased the expression of the epithelial marker E-cadherin and Cytokeratin 18 in ZR-75-30 cells, whereas DACH1 knockdown reduced the expression of E-cadherin and Cytokeratin 18 in MCF-7 and T47D cells (Figures 2h and i). For the mesenchymal markers N-cadherin and Vimentin, DACH1 overexpression effectively suppressed their expression levels in ZR-75-30 cells, whereas DACH1 silencing increased their expression levels in MCF-7 and T47D cells (Figures 2h and i). Furthermore, we performed immunofluorescence assay to detect the localization and expression levels of E-cadherin and N-cadherin. With ZR-75-30 cells, DACH1 overexpression increased the expression of E-cadherin as well as its localization to the membrane, but decreased the expression of N-cadherin (Figure 2j). In contrast, depletion of DACH1 in MCF-7 and T47D cells greatly reduced the expression and membrane localization of E-cadherin, but enhanced the expression of N-cadherin (Figure 2k). Taken together, these results demonstrated that DACH1 might have an important role in regulating the expression of epithelial and mesenchymal markers during EMT in breast cancer cells.

### DACH1 modulates the activity of E-cadherin promoter and the levels of E-cadherin mRNA

**Figure 3:**
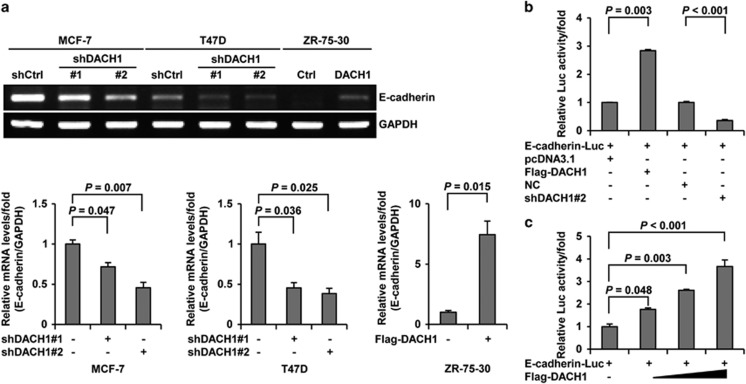
DACH1 regulates E-cadherin mRNA levels and promoter activity. (**a**) RT-PCR showing the effects of DACH1 overexpression and knockdown on the mRNA levels of E-cadherin in ZR-75-30, MCF-7 and T47D cells (top). The bar graph shows the fold changes in relative mRNA levels normalized against GAPDH (bottom). ZR-75-30 cells were transfected with pcDNA3.1-3 × Flag-DACH1 or pcDNA3.1-3 × Flag vector; MCF-7 and T47D cells were transfected with shDACH1#1, shDACH1#2 and shCtrl (pRNAT-U6.1 vector). (**b** and **c**) Reporter-gene assay showing the regulation of E-cadherin luciferase reporter activity by DACH1 in MCF-7 cells. Cells were cotransfected with E-cadherin-Luc and DACH1, shDACH1#2 (pRNAT-U6.1 vector) or empty vector, and then cultured for 48 h before harvest. The bar graph shows the fold changes in relative luciferase activity normalized against *β*-galactosidase activity. Data are presented as means±s.d.s. Significance was determined using the Bonferroni test (*P*<0.05, significant ns, not significant).

As DACH1 could increase the expression of E-cadherin (Figure 2h), it became important to know the underlying mechanism by which this was accomplished. Reverse transcription-PCR (RT-PCR) analysis of E-cadherin mRNA level in ZR-75-30 cells that overexpressed DACH1 revealed a significant increase compared with the level of E-cadherin mRNA of control cells (Figure 3a). In contrast, the levels of E-cadherin mRNA in MCF-7 and T47D cells in which shDACH1 had been knocked down were greatly reduced compared with the level of E-cadherin mRNA in control cells (Figure 3a). This clearly indicated that E-cadherin transcription was subjected to regulation by DACH1. Reporter-gene assay also showed that the level of E-cadherin-driven reporter activity in the cells increased when the cells overexpressed DACH1 (Figures 3b and c) but decreased when DACH1 was knocked down in the cells (Figure 3b). These observations indicated that DACH1 promoted E-cadherin transcription, resulting in its increased mRNA level and activity.

### DACH1 binds to the E-box region of the E-cadherin promoter

**Figure 4:**
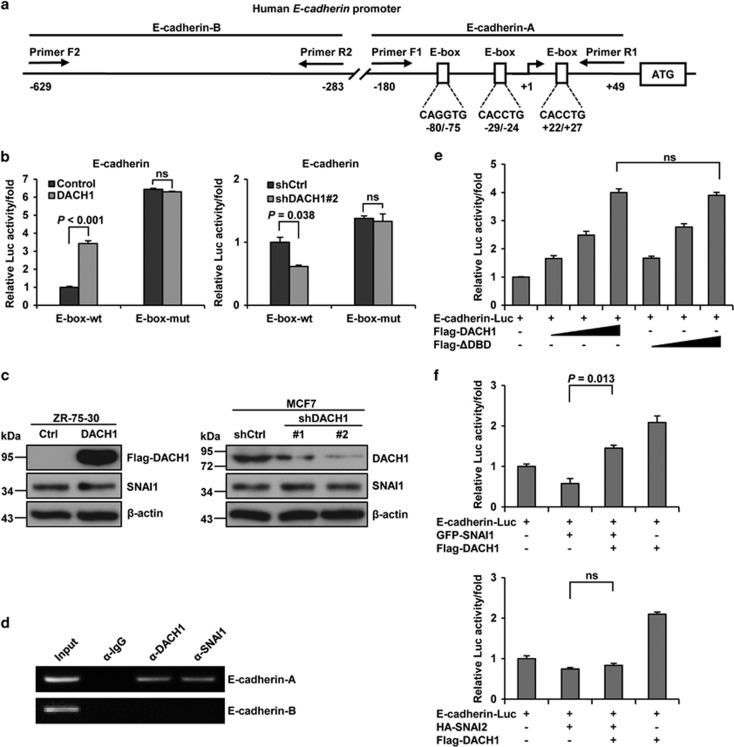
DACH1 is recruited to E-cadherin in an E-box-dependent and DBD-independent manner in MCF-7 cells. (**a**) Schematic illustration of the E-box elements and chromatin-immunoprecipitation (ChIP) region in the E-cadherin promoter. The E-cadherin-A fragment includes three E-box elements and the E-cadherin-B fragment as control. (**b**) Reporter-gene assay showing the regulation of luciferase reporter activity by DACH1 overexpression (left) or knockdown (right) in MCF-7 cells. Luciferase activity was driven by wild-type E-box or mutant E-box containing E-cadherin promoter. Cells were cotransfected with E-box-wt-Luc or E-box-mut-Luc and DACH1, shDACH1#2 or empty vector, and then cultured for 48 h before harvest. (**c**) Western blots showing the effects of DACH1 overexpression and knockdown on the expression levels of SNAI1 in ZR-75-30 and MCF-7 cells. (**d**) ChIP assay showing the interaction between DACH1 or SNAI1 and the E-box region (E-cadherin-A shown in Figure 4a) of E-cadherin promoter in MCF-7 cells. Cross-linked chromatin was extracted from the cells and subjected to immunoprecipitation with antibody against DACH1, SNAI1 or IgG, and the DNA was used for amplification of E-cadherin-A and -B fragments shown in (**a**). (**e**) Reporter-gene experiments showing the regulation of luciferase reporter activity of E-cadherin by DACH1 or ΔDBD in MCF-7 cells. Cells were cotransfected with E-cadherin-Luc and DACH1, ΔDBD or empty vector. (**f**) Reporter-gene assay showing the regulation of E-cadherin-luciferase activity by DACH1 and SNAI1 overexpression (top) or SNAI2 overexpression (bottom) in MCF-7 cells. Cells were cotransfected with E-cadherin-Luc, DACH1 and SNAI1 or SNAI2. All reporter gene bar graphs show the fold change of relative luciferase activity normalized to *β*-galactosidase activity. Data are presented as means±s.d.s. Significance was determined using the Bonferroni test (*P*<0.05, significant ns, not significant).

E-cadherin transcription has previously been shown to be directly repressed by EMT transcription factors that bind to the E-box in its promoter region (Figure 4a).^10, 41^ Thus, the regulation of E-cadherin expression by DACH1 may also be E-box dependent. To test this hypothesis, the 5′-CAGGTG-3′ or 5′-CACCTG-3′ region of three E-boxes of the E-cadherin promoter was mutated to 5′-AACCTA-3′, a sequence that was unable to bind EMT transcription factors. Interestingly, overexpression of DACH1 could not upregulate the activity of E-box-mut compared with E-box-wt (Figure 4b, left). Moreover, shDACH1 also could not downregulate the activity of E-box-mut compared with E-box-wt (Figure 4b, right). These findings suggested that DACH1 increased the activity of E-cadherin promoter in an E-box-dependent manner.

Given that E-cadherin is regulated by SNAI1,^20^ and SNAI1 has been shown to be indirectly regulated by DACH1,^40^ we sought to determine whether E-cadherin is regulated by DACH1 through SNAI1. Surprisingly, the expression of SNAI1 did not change, neither in ZR-75-30 cells transfected with DACH1 nor in MCF-7 cells transfected with shDACH1 (Figure 4c). In order to obtain further insight into the mechanism by which DACH1 enhances E-cadherin activity, chromatin-immunoprecipitation (ChIP) assay was used to analyse the E-box region of the E-cadherin promoter that may interact with DACH1. The result showed that DACH1 specifically interacted with the E-cadherin-A fragment, which included three E-box elements (Figure 4d, top). We also amplified the E-cadherin-B fragment as a control (Figure 4d, bottom). This suggested that regulation of E-cadherin activity was dependent on the DNA-binding domain (DBD) of DACH1. To test this, a similar reporter gene assay was conducted, but, instead of wild-type DACH1, a mutant DACH1 having a deletion at the DBD (DACH1-ΔDBD) was overexpressed. Unexpectedly, the level of E-cadherin-Luc activity was even higher when the cells overexpressed the mutant DACH1 (Figure 4e). This result demonstrated that the DBD was not important in the regulation of E-cadherin, suggesting that DACH1 did not directly interact with the E-box region. Therefore, we speculated that other factors may be involved in coordinating the interaction between DACH1 and the E-box of E-cadherin. A further test was carried out using different EMT zinc-finger transcription factors, such as SNAI1 and SNAI2, which are known to repress E-cadherin through direct binding with the E-box.^6, 10, 20, 42^ Overexpression of SNAI1 or SNAI2 alone repressed E-cadherin-Luc activity, whereas overexpression of SNAI1 and DACH1 relieved this repression and overexpression of SNAI2 and DACH1 had no effect on the SNAI2-repressed E-cadherin-Luc activity (Figure 4f). Taken together, these findings revealed that DACH1 may enhance the activity of E-cadherin by alleviating its transcriptional suppression exerted by SNAI1.

### DACH1 interacts with SNAI1 to increase the expression of E-cadherin

**Figure 5:**
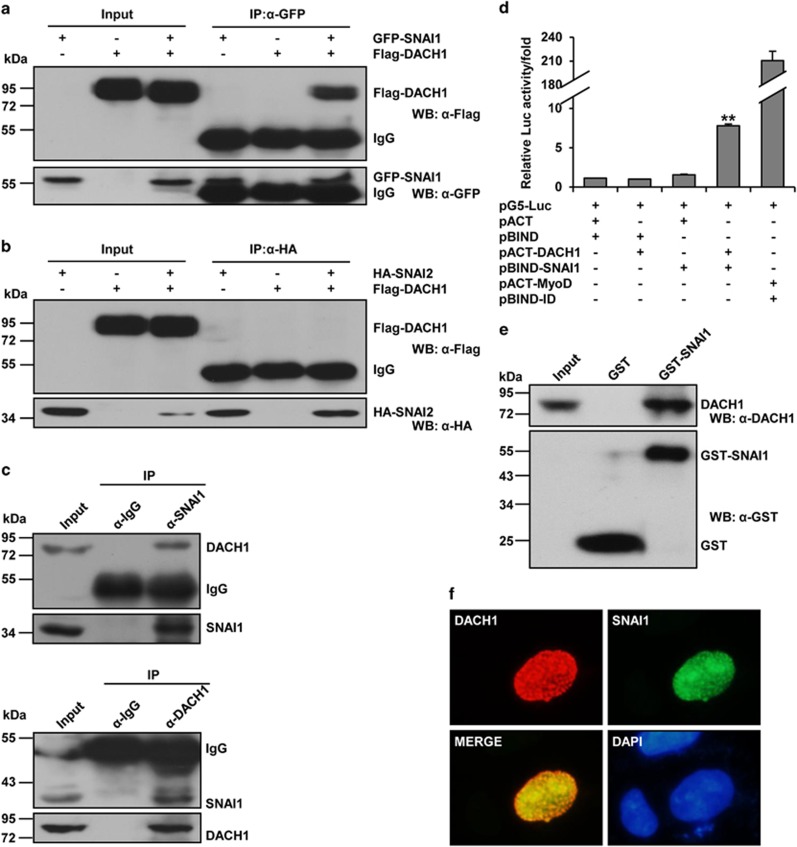
Evidence for the interaction between DACH1 and SNAI1 in MCF-7 cells. (**a** and **b**) CoIP assay showing the interaction between exogenous DACH1 and SNAI1 and between exogenous DACH1 and SNAI2. MCF-7 cells were cotransfected with Flag-DACH1 and GFP-SNAI1 or HA-SNAI2. After 24 h of transfection, the cells were subjected to immunoprecipitation with anti-Flag antibody or anti-HA antibody, followed by western blot analysis with anti-GFP antibody. (**c**) CoIP assay showing the interaction between endogenous DACH1 and SNAI1. MCF-7 cells were subjected to immunoprecipitation with anti-SNAI antibody or anti-DACH1 antibody and anti-IgG antibody, followed by western blot analysis with anti-DACH1 antibody or anti-SNAI antibody. (**d**) Mammalian two-hybrid assay analysis of the interaction between DACH1 and SNAI1 in MCF-7 cells. Cells were cotransfected with either pACT-DACH1 and pBIND-SNAI1, empty vectors pACT and pBIND (negative control), or pBIND-ID and pACT-MyoD (positive control). Luciferase activity was measured after 48 h of transfection. The bar graph shows the fold change of relative luciferase activity normalized against *β*-galactosidase. **P<0.01 compared with cells transfected with pACT and pBIND. (**e**) GST pulldown assay showing the interaction between GST-SNAI1 and DACH1. (**f**) Immunofluorescence showing the colocalization of DACH1 (red) and SNAI1 (green) in the nucleus of MCF-7 cells. Cells were grown on coverslips and cotransfected with Flag-DACH1 and GFP-SNAI1. After 24 h, cells were fixed and immunostained with anti-Flag antibodies and then incubated with secondary antibodies (Alexa Fluor 568). Nuclear proteins were stained with DAPI.

In view of the important participation of DACH1 and SNAI1 in the regulation of E-cadherin (Figures 4d and f), we sought to determine whether DACH1 and SNAI1 could form a complex at the E-cadherin promoter. Co-immunoprecipitation (CoIP) assay was used to test the interaction between DACH1 and SNAI1, and the result showed that DACH1 specifically interacted with SNAI1 in MCF-7 cells (Figure 5a) but did not interact with SNAI2 (Figure 5b). To further extend our finding to MCF-7 cells, the association between endogenous DACH1 and SNAI1 was established (Figure 5c). In addition, the mammalian two-hybrid system further confirmed the direct interaction between DACH1 and SNAI1 (Figure 5d). Glutathione S-transferase (GST) pulldown assays confirmed the direct interaction between GST-SNAI1 and DACH1 (Figure 5e). Furthermore, immunofluorescence analysis revealed that DACH1 and SNAI1 were colocalized in the nucleus in MCF-7 cells (Figure 5f).

**Figure 6:**
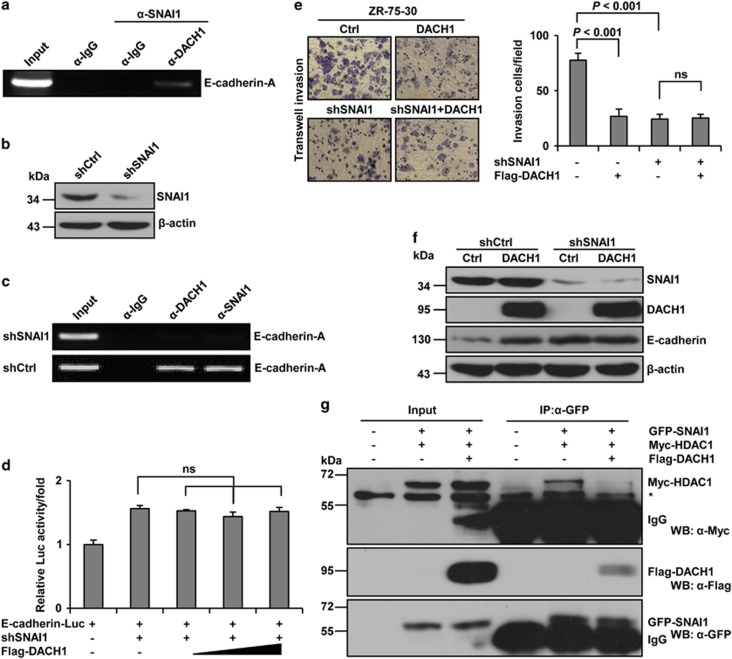
DACH1 is recruited by SNAI1 to the E-cadherin promoter. (**a**) ChIP-reChIP experiments showing the binding of DACH1 and SNAI1 to the E-box (Fragment-A shown in Figure 4a) of the E-cadherin promoter in MCF-7 cells. DNA–protein complexes were first subjected to immunoprecipitation with anti-SNAI1 antibody, followed by immunoprecipitation with anti-DACH1 antibody as a second round of ChIP. (**b**) Western blot analysis of SNAI1 knockdown in MCF-7 cells. (**c**) ChIP experiments investigating the interaction between DACH1 or SNAI1 and the E-box region (Fragment-A shown in Figure 4a) of the E-cadherin promoter in MCF-7 cells in which SNAI1 had been knocked down. (**d**) Reporter-gene assay showing the effect of DACH1 on E-cadherin-luciferase activity in MCF-7 cells. Cells were cotransfected with E-cadherin-Luc, DACH1 and shSNAI1 and then incubated for 48 h before harvest. The bar graph shows the fold change of relative luciferase activity normalized against *β*-galactosidase activity. (**e**) Transwell invasion assays evaluating the effect of DACH1 overexpression on the cellular invasion ability of SNAI1-knockdown ZR-75-30 cells. Cell invasion assay was performed in 24-well chambers with Matrigel. Cells (1 × 10^5^ per well) were transferred to the upper chamber. After 24 h of incubation, the invading cells on the lower surface of the filter were stained and counted (left). The bar graphs show the number of migrating and invading cells for each category of cells (right). (**f**) Western blots showing the effects of DACH1 overexpression on the expression levels of E-cadherin in SNAI1-knockdown ZR-75-30 cells. (**g**) CoIP assay showing the effect of DACH1 on the interaction between DACH1 and SNAI1. MCF-7 cells were cotransfected with Flag-DACH1, GFP-SNAI1 or Myc-HDAC1. *, nonspecific band. Data are presented as means±s.d.s. Significance was determined using the Bonferroni test (*P*<0.05, significant ns, not significant).

ChIP-reChIP showed that DACH1 and SNAI1 were bound to the same regions on the E-box of the E-cadherin promoter (Figure 6a). The recruitment of DACH1 to the E-cadherin promoter was dependent on the presence of SNAI1, as knockdown of SNAI1, which led to a reduced level of SNAI1 protein (Figure 6b), effectively prevented the recruitment of DACH1 to the promoter of E-cadherin (Figure 6c). Furthermore, knockdown of SNAI1 did not alter the level of E-cadherin-Luc reporter activity seen with SNAI1 knockdown alone (Figure 6d). In addition, DACH1 overexpression did not change the invasiveness or the expression of E-cadherin in SNAI1-knockdown ZR-75-30 cells (Figures 6e and f), indicating that the effect of DACH1 on the expression of E-cadherin was mediated by SNAI1. HDAC1 has been demonstrated to interact with SNAI1 and inhibit the expression of E-cadherin as a corepressor of SNAI1;^21^ therefore, we sought to determine whether DACH1 can affect the interaction between SNAI1 and HDAC1. Overexpression of DACH1 markedly reduced the interaction between SNAI1 and HDAC1 (Figure 6g). Taken together, our results showed that DACH1 interacted with SNAI1 and inhibited the recruitment of HDAC1 to SNAI1, effectively suppressing the transcriptional activity of SNAI1 and eventually leading to the activation of E-cadherin.

### DACH1 reduces breast cancer cell metastasis *in vivo*

**Figure 7:**
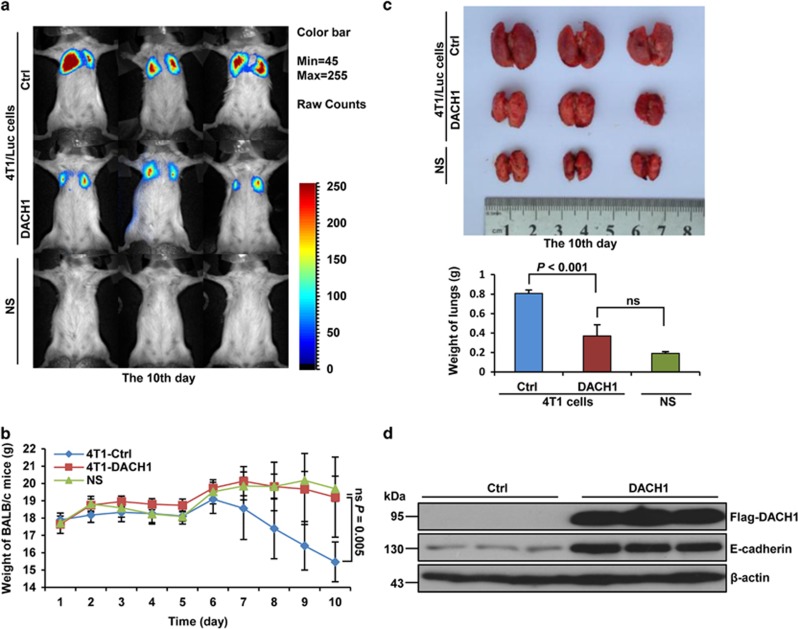
DACH1 modulates the metastasis and growth of 4T1/Luc cells in BALB/c mice. (**a**) Bioluminescent images showing the effect of DACH1 overexpression on breast cancer cells. Three representative BALB/c mice from each group are shown. BALB/c mice were inoculated with 4T1/Luc cells or with normal saline (NS) by tail vein injection. 4T1/Luc cells were transfected with pcDNA3.1-3 × Flag-DACH1 or pcDNA3.1-3 × Flag vector and grown for 5 days in the presence of G418. Ten days after the tail vein injection, firefly luciferase bioluminescence signals of the 4T1/Luc cells were acquired and analysed using the CRi Maestro *In vivo* Imaging System. Before imaging, D-luciferin was injected into the peritoneal cavity of the mice as the firefly luciferase substrate. (**b**) Line graph image showing the effect of DACH1 overexpression on the weight of BALB/c mice. The weights of BALB/c mice used in (**a**) were evaluated every day. (**c**) The images showing the effect of DACH1 overexpression on lung size in mice (left). The bar graph shows the quantitative measure of the weight of lung in mice (right). (**d**) Western blots showing the effects of DACH1 overexpression on the expression levels of E-cadherin in the lung carcinoma tissues from three representative BALB/c mice used in (**a**). Data are presented as means±s.d.s (*n*=6). Significance was determined using the Bonferroni test (*P*<0.05, significant ns, not significant).

To extend our studies to an animal metastasis model, we examined whether the levels of DACH1 might correlate with metastasis in BALB/c mice. BALB/c mice injected with 4T1/Luc cells that stably overexpressed DACH1 showed significant reduction of lung metastasis, compared with mice injected with 4T1/Luc that stably expressed the firefly luciferase (positive control) or those injected with just normal saline (negative control) (Figure 7a). Furthermore, mice that overexpressed DACH1 showed no reduction in weight but maintained a similar trend in weight gain as the negative controls, whereas the positive controls suffered significant weight loss from day 6 onwards, and the weight loss trend continued until the end of the experiment (Figure 7b). The sizes and weights of the lungs of BALB/c mice that overexpressed DACH1 were also significantly decreased compared with positive controls (Figure 7c), but were still bigger than those of negative controls. As for the changes in E-cadherin protein level, 4T1/Luc cells taken from the lung tissue showed a much higher level of E-cadhrein protein when these cells overexpressed DACH1 (Figure 7d). Taken together, these data showed that DACH1 could inhibit breast cancer metastasis in our animal model, thus supporting our finding that DACH1 acts as a crucial factor in the suppression of metastasis of breast cancer cells.

**Figure 8:**
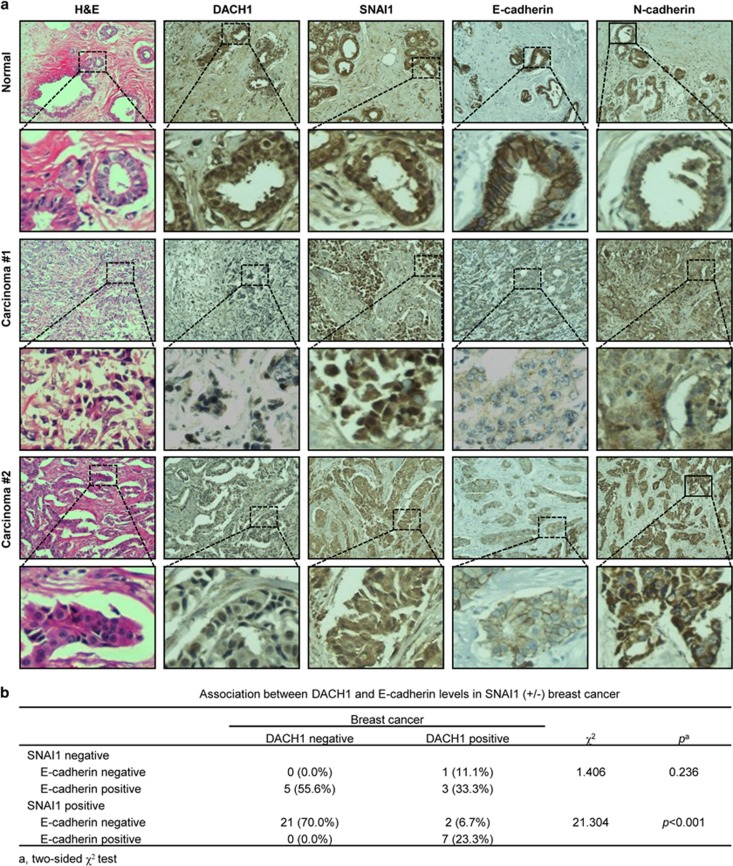
Correlation between DACH1 and E-cadherin expressions in human breast tissue samples. (**a**) Representative examples of immunohistochemical staining for DACH1, SNAI1, E-cadherin and N-cadherin in normal human breast tissues (*n*=5) and breast carcinoma tissues (*n*=39) as indicated. (**b**) Association between DACH1 and E-cadherin levels in SNAI1 (+/−) breast cancer tissue samples. Significance was determined using a two-sided *χ*^2^ test (*P*<0.05).

To further determine the *in vivo* relevance of our data obtained with cell culture, the expression levels of DACH1, SNAI1, E-cadherin and N-cadherin of five normal human breast tissues and 39 breast carcinoma tissues were analysed. DACH1 was expressed in normal breast tissues with reduced expression in breast cancer tissues (Figure 8a). Moreover, changes in the level of E-cadherin were also consistent with the level of DACH1. We also analysed the association between DACH1 and E-cadherin levels in SNAI1-positive or -negative breast cancer tissue samples (Figure 8b). Significant correlation was observed between DACH1 and E-cadherin in SNAI1-positive samples (Figure 8b). These findings were consistent with our speculation that DACH1 interacted with SNAI1 to decrease the transcriptional activity of SNAI1, consequently increasing the expression of E-cadherin.

## Discussion

DACH1 has been implicated in the suppression of tumour metastasis by binding to a subset of genes and repressing their expression.^28^ A previous report has demonstrated that DACH1 inhibits cellular migration and invasion of breast cancer by binding to and repressing the *IL-8* gene.^38^ Recently, DACH1 has also been shown to inhibit EMT through repression of the cytoplasmic translation of SNAI1 by inactivating the Y box-binding protein (YB-1).^40^ Inactivation of YB-1 also results in suppression of the YB-1-mediated gene expression that governs cell invasion. These functions of DACH1 have shed more light on the mechanism by which DACH1 acts as a tumour suppressor. In this study, we showed that in breast cancer cells the expression of DACH1 was specifically associated with cell adhesion. DACH1 also regulated the migration and invasiveness of breast cancer cells, a function that appeared to be independent of its general effects on cell proliferation. Our data did confirm that DACH1 acts as a negative regulator of EMT by showing that DACH1 expression effectively inhibited the cell–cell adhesion and motility of breast cancer cells. E-cadherin, the cellular adhesion molecule that forms the cell–cell adhesion junctions of epithelial cells, is essential for the cells to maintain their epithelial phenotype.^12^ During the onset of EMT, E-cadherin is cleaved at the plasma membrane and subsequently degraded, resulting in loss of epithelial adherent junctions.^13^ Thus, downregulation or loss of E-cadherin expression is considered to be the hallmark event of EMT.^3, 6, 13^ In support of this mechanism, increase in E-cadherin expression will effectively increase cell–cell adhesion and reduce cell motility in tumour cells. Such speculation was supported by our data, which showed that the expression of E-cadherin paralleled the expression of DACH1 (Figures 1e and f) and that DACH1-mediated enhanced transcription of E-cadherin in breast cancer cells ultimately led to the inhibition of EMT in these cells (Figure 3). Previous reports have shown that E-box is required for members of the EMT transcription factor that repress E-cadherin transcription.^10^ Our results demonstrated that E-box was also required for DACH1-mediated increased reporter activity of E-cadherin (Figures 4b and d), thus confirming the essential role of E-box in the regulation of E-cadherin by DACH1. The downregulation of E-cadherin would lead to induction of other mesenchymal markers such as N-cadherin during EMT, and this would then activate a ‘cadherin switch' that alters cell–cell contact.^5, 43^ Overexpression of DACH1 increased the expression of E-cadherin, and at the same time also reduced the expression of N-cadherin (Figure 2h). Among the EMT transcription factors, *TWIST1* is the only gene that exhibits any interaction with E-box. It binds to the E-box of the E-cadherin promoter,^44^ as well as to the E-box of the N-cadherin promoter to regulate the activity of downstream target genes.^45^ Whether DACH1 regulates the expression of N-cadherin by binding to the E-box of the promoter requires further study.

Given that the regulation of mesenchymal markers is crucial to EMT,^11, 13^ the effect of DACH1 expression on the expression levels of another epithelial marker and two mesenchymal markers in breast cancer cells was investigated. In addition to the upregulation of E-cadherin expression, overexpression of DACH1 resulted in increased expression of Cytokeratin 18 but decreased expression of N-cadherin and Vimentin (Figure 2h), further supporting the role of DACH1 in the regulation of EMT. As E-cadherin expression has previously been shown to be downregulated by SNAI1,^20^ which in turn is downregulated by DACH1 at the translation level via YB-1,^40^ we speculated that DACH1 might indeed upregulate the expression of E-cadherin by suppressing SNAI1 translation. However, contrary to previous findings that showed a suppression of SNAI1 translation by DACH1 expression, we did not observe any change in the level of SNAI1 protein upon DACH1 overexpression (or silencing). The reason is unclear at this stage, but could be due to the different cell lines used: ZR-75-30 and MCF-7 cells in our case, and MDA-MB-231 cells in the previous study.^40^ This prompted us to look for an alternative way in which DACH1 could regulate E-cadherin through SNAI1, and what we found appeared to be a direct interaction between DACH1 and SNAI1, in which the assumed DACH1-SNAI1 complex might bind to the E-cadherin promoter in a SNAI1-dependent manner (Figures 5 and 6), an interaction that consequently led to the upregulation of E-cadherin transcription. A previous study has demonstrated that SNAI1 can suppress the activity of E-cadherin promoter by deacetylating histones H3 and H4, a reaction that is carried out by HDAC1 and HDAC2, both of which are recruited by SNAI1 upon its binding to the E-cadherin promoter.^21^ HDAC1 and HDAC3 also associate with DACH1, as demonstrated by ChIP and CoIP experiments.^33, 46^ DACH1 reduced the interaction between SNAI1 and HDAC1 (Figure 6g), and probably inhibited the recruitment of HDAC1 to SNAI1 by steric-hindrance effects, ultimately leading to the activation of E-cadherin. Taken together, our finding has uncovered another mode of regulation for E-cadherin transcription in breast cancer, one that is based on direct antagonism of the SNAI1 protein by DACH1.

Previous studies have demonstrated that the DBD of DACH1 is essential for the repression of Smad4, c-Jun and AR activities,^33, 36, 46^ but is not required for the repression of ERα and p53 activities by DACH1.^34, 47^ In addition, DACH1 can directly suppress IL-8 by binding to the *IL-8* promoter via the AP-1 and NF-κB binding sites on the *IL-8* promoter.^38^ DACH1 also suppresses FOXM1-targeted gene expression by binding to the FOXM1 binding site in a competitive mode.^39^ Our data suggested that binding between DACH1 and E-cadherin promoter was not necessary as deletion of the DBD of DACH1 did not change the level of E-cadherin-driven report activity as seen with wild-type DACH1 (Figure 4e). SNAI1 and SNAI2 have been shown to directly bind to the E-box of the E-cadherin promoter.^6, 10, 20, 42^ Therefore, DACH1 probably abolished the SNAI1-mediated suppression of E-cadherin transcription (Figure 6f) probably through direct binding with SNAI1 and altering its affinity for the E-cadherin promoter. Although SNAI2 is highly related and homologous to SNAI1, no interaction between DACH1 and SNAI2 was detected in our study (Figure 5b). Therefore, we suspected that DACH1 may interact with the non-homologous region of SNAI1 and SNAI2, such as the nuclear export signal and destruction box.^10^ Further investigation is needed to clarify this issue. In addition to SNAI1 and SNAI2, we could not exclude the possibility that DACH1 might interact with other EMT factors, such as ZEB1, ZEB2 and TWIST1.

Metastasis of the cancer is the major cause of death among breast cancer patients. Once the cancer reaches the stage of metastasis, the disease becomes difficult to control.^1, 3^ Therefore, the hunt for therapeutic agents that target the metastasis of breast cancer has become an intractable goal. Although we have shown here an alternative pathway by which DACH1 regulates SNAI1 to bring about suppression of EMT and metastasis in breast cancer, the exact domains of DACH1 and SNAI1 involved in the DACH1–SNAI1 interaction, and whether there are other molecules involved in the interaction between DACH1 and SNAI1, remain unknown at this stage. Hopefully, better insights into the regulation of DACH1-mediated repression of cancer metastasis from future investigations may form the basis for designing better therapies that specifically target the metastatic stage of breast cancer.

## Materials and methods

### Cell culture and transfection

MCF-7 and T47D cells were cultured as previously described.^48, 49^ ZR-75-30 and 4T1/Luc (stable transfection of the firefly luciferase gene) cells were generously provided by Dr Yongli Bo (Northeast Normal University, China) and were cultured in RPMI-1640 medium. Cells were grown at 37 ° C in a humidified 5% CO_2_ atmosphere and transfected using Lipofectamine 2000 (Invitrogen, Auckland, New Zealand) according to the manufacturer's specifications.

### Plasmids and antibodies

The pcDNA3.1-3 × Flag vector was gifted by Dr Shigeru Takahashi (Tokyo University of Pharmacy and Life Sciences, Japan). pKW-Flag-DACH1 and pKW-Flag-DACH1-ΔDBD plasmids were gifts from Dr Richard G. Pestell (Thomas Jefferson University, USA). pcDNA3.1-3 × Flag-DACH1 and pcDNA3.1-3 × Flag-DACH1-ΔDBD were generated using the above construct as a template. The sequences of primers are as follows: 5′-CGGGATCCATGGCAGTGCCGGCGGCTTT-3′ (forward) and 5′-CCGCTCGAGTCAGTACATGACAGTAGTT-3′ (reverse). pEGFP-SNAI1, pcDNA3.1-HA-SNAI2, NC (shCtrl), shSNAI1, pGL3 vector and pGL3-E-cadherin-Luc were acquired as previously described.^5, 50^ For plasmids of the Checkmate Mammalian Two-Hybrid System (Promega, Madison, WI, USA), DACH1 and SNAI1 were subcloned into *Bam*HI-*Eco*RV cut pACT and pBIND, respectively. Mutant promoters in the three E-boxes of E-cadherin were obtained using the site-directed gene mutagenesis kit (Beyotime, Jiangsu, China). DACH1 shRNA expression vectors, the pRNAT-U6.1 vector (GenScript, Piscataway, NJ, USA), shDACH1#1 and shDACH1#2 were used for DACH1 knockdown study, and the sequences were as follows: 5′-AAAGTGGCTTCCTTTACGGTG-3′^46^ and 5′-GCACTTGAGTTTGAGACGA-3′.^51^

Rabbit anti-Flag, anti-GFP, anti-HA and mouse anti-Flag antibodies were purchased from Sigma (Sigma, Saint Louis, MO, USA). Mouse anti-E-cadherin, anti-Cytokeratin 18 and rabbit anti-Vimentin antibody were obtained from Abcam (Abcam, Cambridge, MA, USA). Rabbit anti-N-cadherin, anti-DACH1 and anti-SNAI1 antibodies were purchased from Santa Cruz Biotechnology (Santa Cruz, Dallas, CA, USA).

### Immunofluorescence and immunohistochemistry

Immunofluorescence and immunohistochemistry were performed as previously described.^5, 52^ The slides were incubated with antibody directed against DACH1, SNAI1, E-cadherin or N-cadherin. For F-actin staining, the cells were fixed in phosphate buffered saline containing 3.7% formaldehyde for 5 min and then in phosphate buffered saline containing 0.1% Triton X-100 for 10 min, followed by incubation with rhodamine-phalloidin (Sigma) for 40 min at room temperature. Thirty-nine breast carcinoma tissue samples and five normal/pericarcinomatous tissue samples used for immunohistochemical analysis were obtained from Qiqihar Medical University. All individuals who donated the tissues for this study gave their consent in written form.

### Western blot, CoIP and ChIP

Western blot, CoIP and ChIP were conducted as previously described.^49, 52, 53^ For ChIP-reChIP, the chromatin immunocomplexes were subjected to immunoprecipitation with another specific antibody. The primers used in the ChIP PCR analysis were 5′-ACTCCAGGCTAGAGGGTCACC-3′ (forward) and 5′-TGAACTGACTTCCGCAAGCTC-3′ (reverse) for E-cadherin-A (−180/+49) and 5′-AGCACTTTGGGAGGCCAAGGC-3′ (forward) and 5′-CTTTTACACTTGGCTGAGTTC-3′ (reverse) for E-cadherin-B (−629/−283).

### GST pulldown assays

GST alone and GST-SNAI1 fusion proteins were expressed in *Escherichia coli* BL21 cells and purified by means of the Pierce GST Spin Purification Kit (Thermo-Pierce, Rockford, IL, USA). GST pulldown assay was performed using a Pierce GST Protein Interaction Pull-Down Kit (Thermo-Pierce). The purified GST-SNAI1 fusion protein (BAIT) was immobilized on the Pierce Spin Column and then preyed DACH1 from MCF-7 cell lysate.

### Reporter-gene, quantitative RT-PCR and MTT assays

Reporter-gene, quantitative RT-PCR and MTT assays were performed as described in our previous study.^49, 54, 55^ Relative luciferase activity was normalized to *β*-galactosidase and shown as fold changes. For quantitative RT-PCR, the primers used were 5′-CTTCGGAGGAGAGCGGTG-3′ (forward) and 5′-CTAGTCGTCCTCGCCGCC-3′ (reverse) for E-cadherin and 5′-TGAAGGTCGGAGTCAACGG-3′ (forward) and 5′-CCTGGAAGATGGTGATGGG-3′ (reverse) for *GAPDH*.

### Transwell, scratch wound-healing and cell colony scattering assays

Transwell, scratch wound-healing and cell colony scattering assays were performed as previously described.^5, 41^ Before they were plated, cells were transfected with appropriate plasmids and selected for several days in the presence of G418 or Hygromycin B. For cell colony scattering assay, the colonies were divided into three categories: compact (>90% cell–cell contact in the colony), loose (50–90% cell–cell contact in the colony) and scattered (<50% cell–cell contact in the colony) contact with neighbouring cells.

### Mice metastasis model

BALB/c mice (6–8 weeks old) were purchased from the Laboratory Animal Center of Dalian Medical University. 4T1/Luc cells were stably transfected with pcDNA3.1-3 × Flag-DACH1 or pcDNA3.1-3 × Flag vector, and 100 μl of these cells (∼2 × 10^6^ cells) suspended in normal saline was injected into the tail vein of the BALB/c mice. Control BALB/c mice were injected with 100 μl normal saline only. Each group consisted of six animals. On the 10th day, firefly luciferase bioluminescence signals of the 4T1/Luc cells were analysed using the CRi Maestro *In vivo* Imaging System (CRi Inc., Woburn, MA, USA). After the bioluminescence analysis, the animals were killed under anaesthesia and the lungs were removed surgically. The sizes and weights of the lungs were recorded, and the carcinoma tissues were then excised, and 10 mg of the tissue was homogenized in 200 μl RIPA buffer A and western blotting was performed as described above.

### Statistical analysis

All animal experiments and immunohistochemical analysis were approved by the Ethics Committee for Biology and Medical Science of Dalian University of Technology. Data are presented as means±s.d.s of at least three independent experiments. Statistical analyses were carried out with one-way Analysis of variance with Bonferroni's multiple-comparison test. Immunohistochemical data were determined by means of the two-sided *χ*^2^ test. Statistical significance was considered at *P*<0.05.
